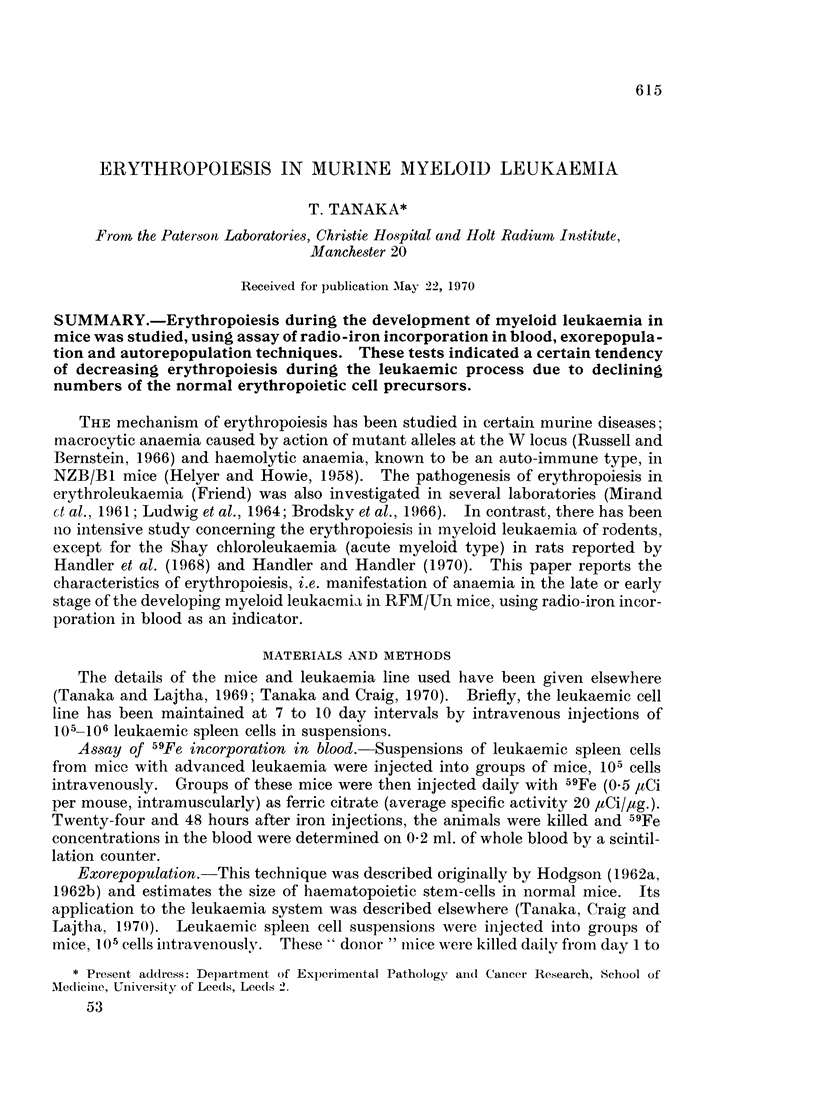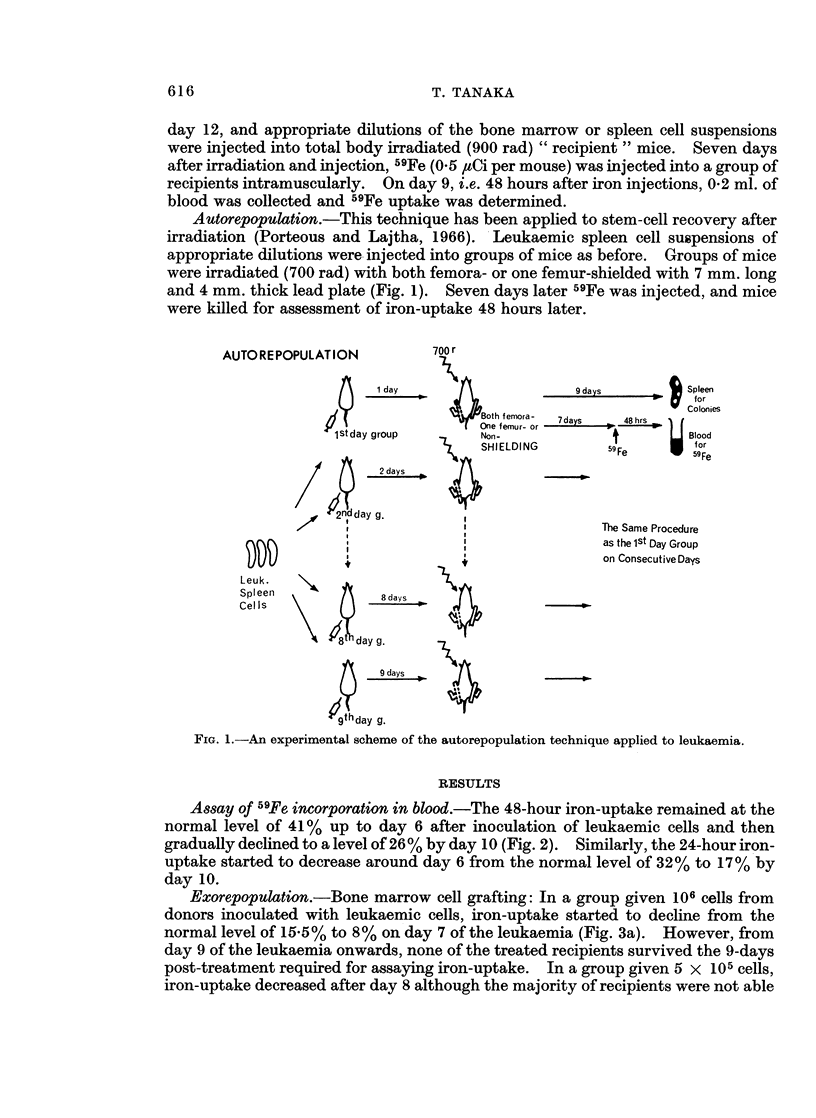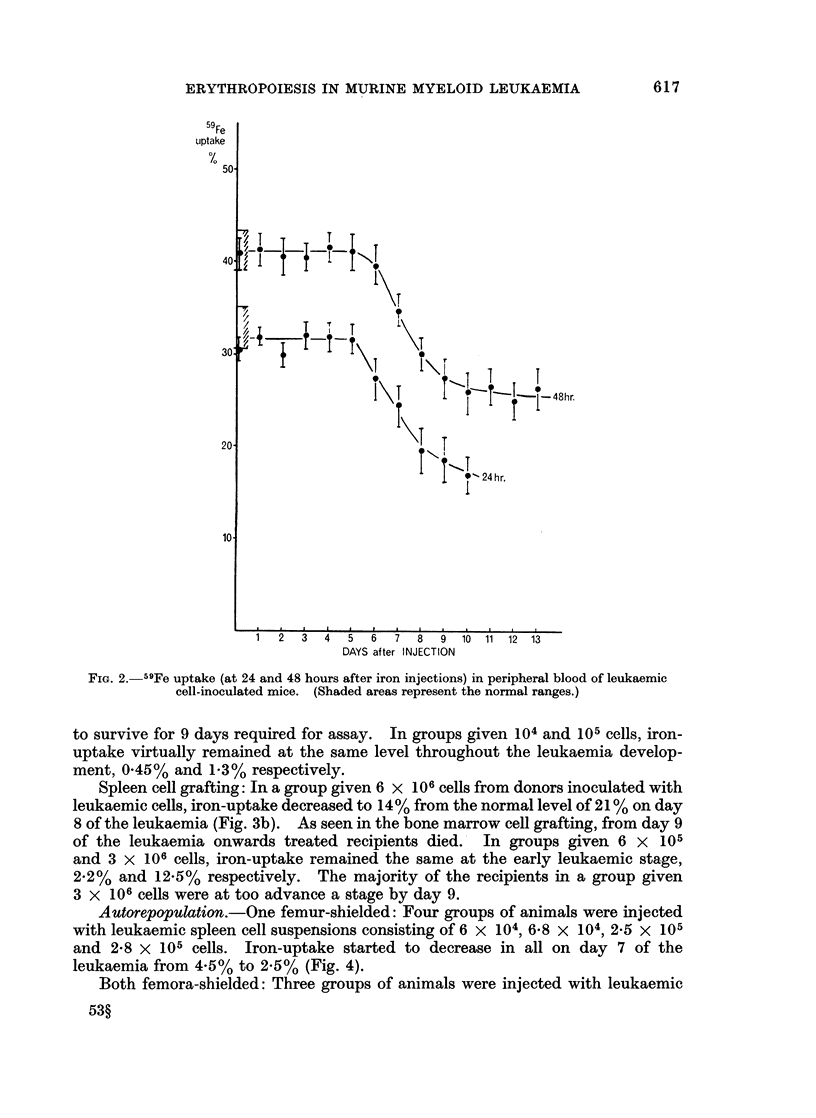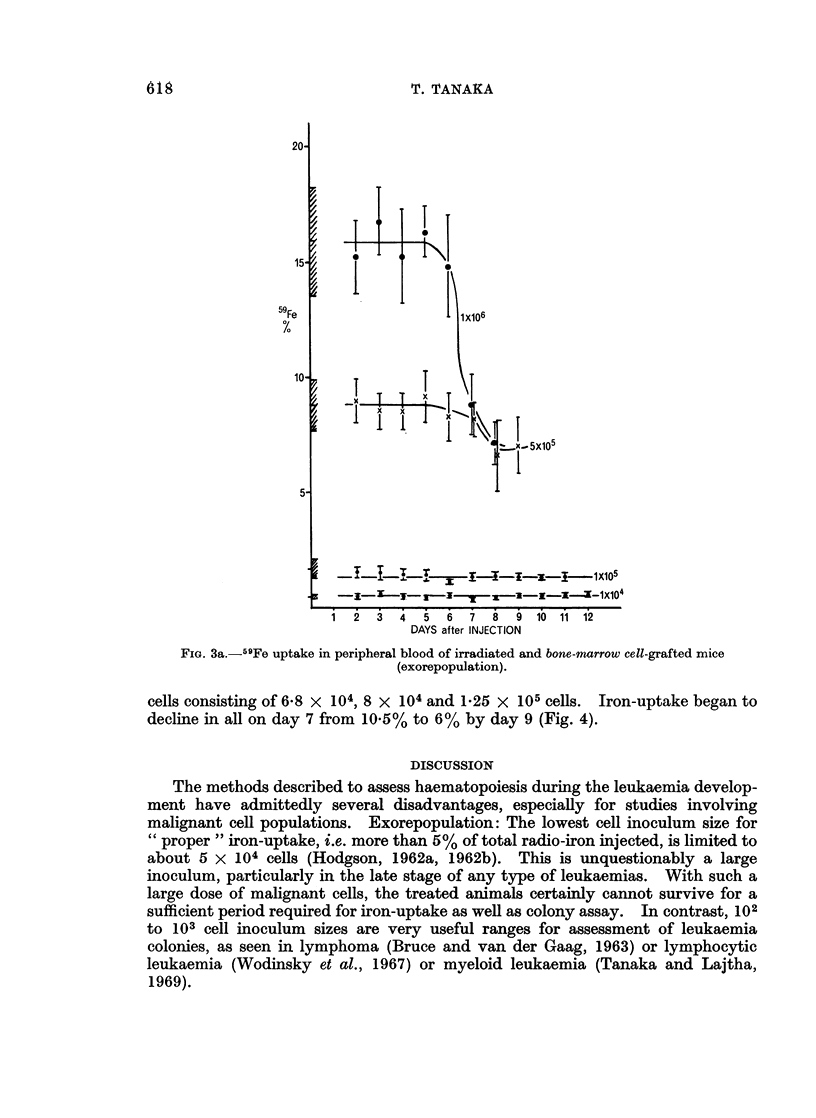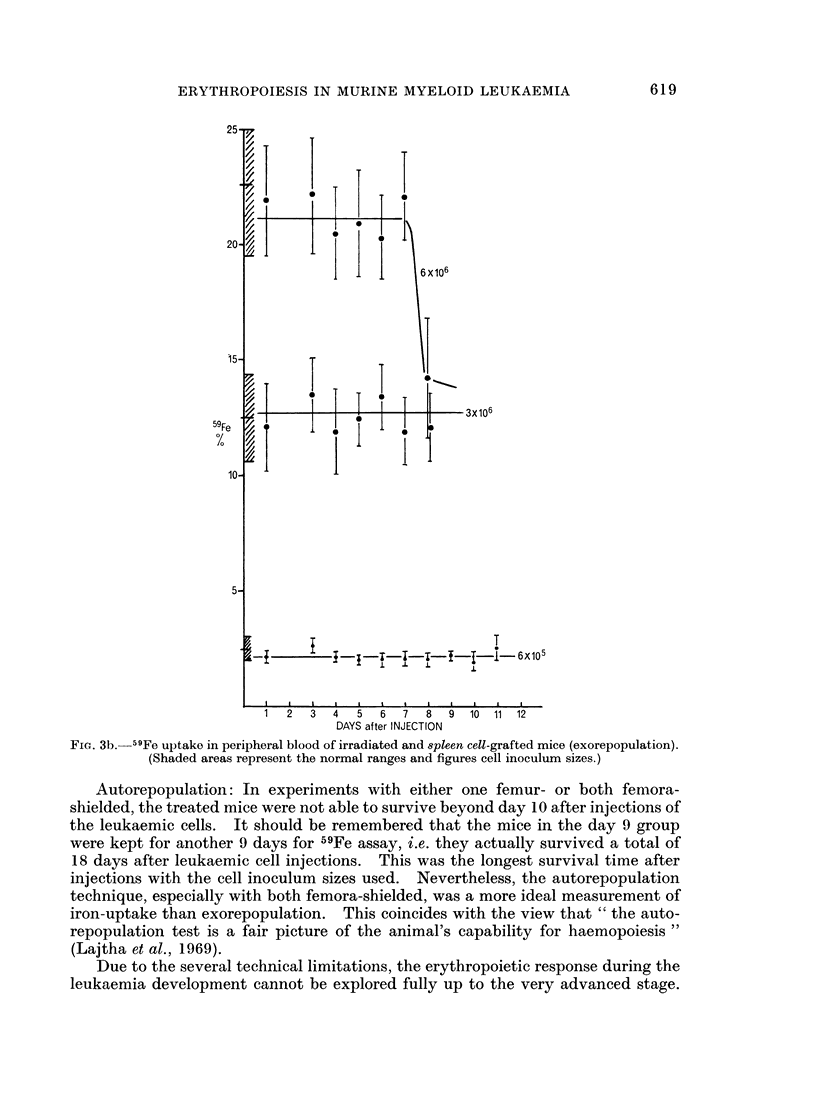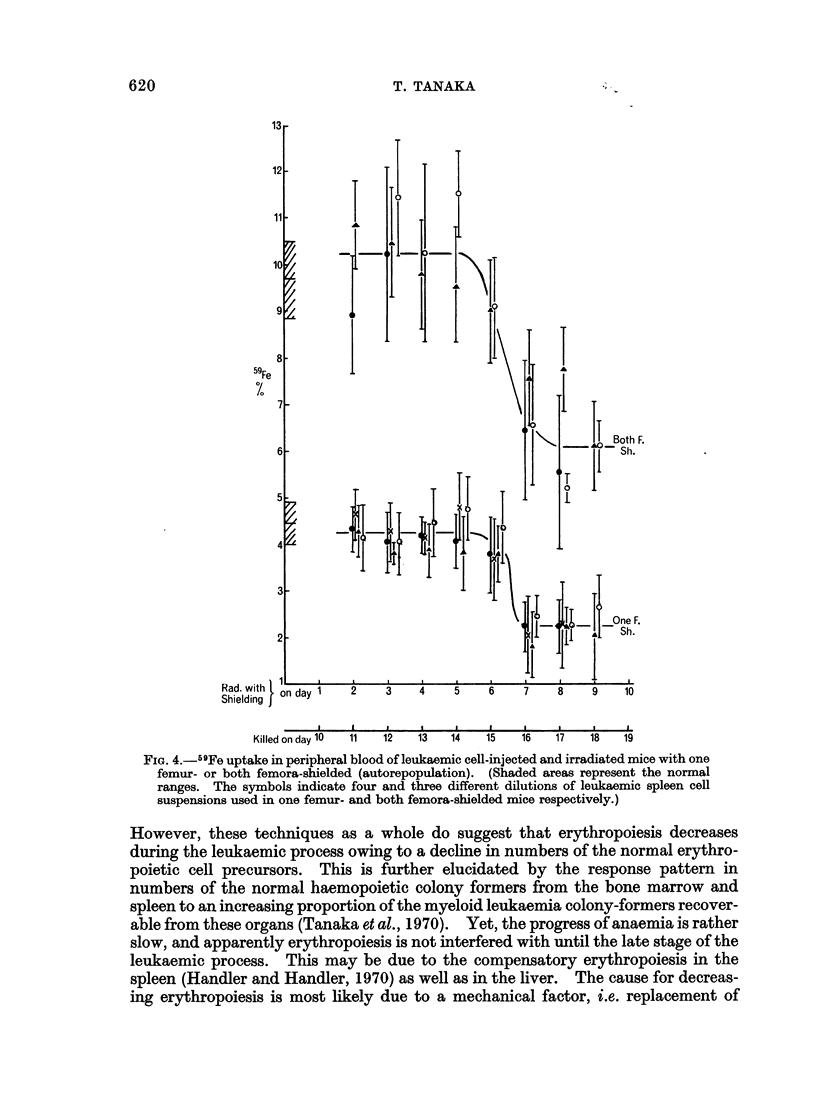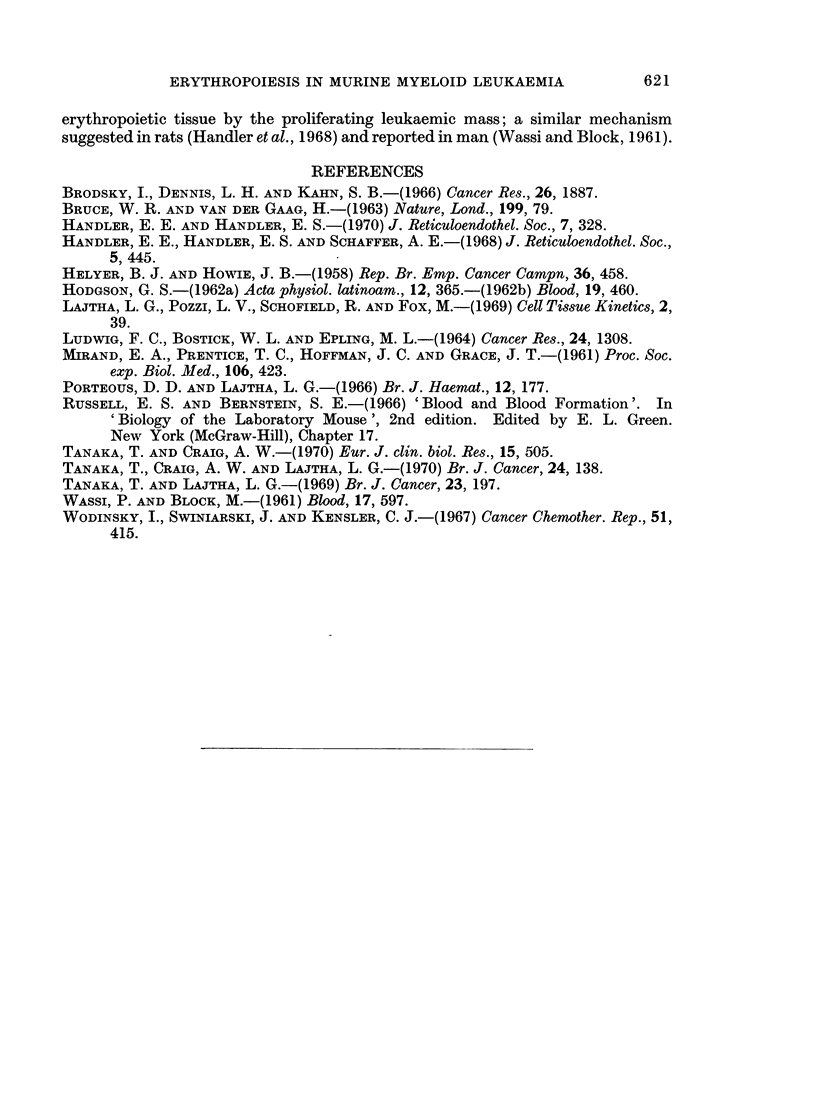# Erythropoiesis in Murine Myeloid Leukaemia

**DOI:** 10.1038/bjc.1970.75

**Published:** 1970-09

**Authors:** T. Tanaka

## Abstract

Erythropoiesis during the development of myeloid leukaemia in mice was studied, using assay of radio-iron incorporation in blood, exorepopulation and autorepopulation techniques. These tests indicated a certain tendency of decreasing erythropoiesis during the leukaemic process due to declining numbers of the normal erthropoietic cell precursors.


### ERYTHROPOIESIS IN MURINE MYELOID LEUKAEMIA

T. TANAKA*

*From the Paterson Laboratories, Christie Hospital and Holt Radium Institute, Manchester* 20

Received for publication May 22, 1970

**SUMMARY.—Erythropoiesis during the development of myeloid leukaemia in mice was studied, using assay of radio-iron incorporation in blood, exorepopulation and autorepopulation techniques. These tests indicated a certain tendency of decreasing erythropoiesis during the leukaemic process due to declining numbers of the normal erythropoietic cell precursors.**

THE mechanism of erythropoiesis has been studied in certain murine diseases; macrocytic anaemia caused by action of mutant alleles at the W locus (Russell and Bernstein, 1966) and haemolytic anaemia, known to be an auto-immune type, in NZB/B1 mice (Helyer and Howie, 1958). The pathogenesis of erythropoiesis in erythroleukaemia (Friend) was also investigated in several laboratories (Mirand *et al.*, 1961; Ludwig *et al.*, 1964; Brodsky *et al.*, 1966). In contrast, there has been no intensive study concerning the erythropoiesis in myeloid leukaemia of rodents, except for the Shay chloroleukaemia (acute myeloid type) in rats reported by Handler *et al.* (1968) and Handler and Handler (1970). This paper reports the characteristics of erythropoiesis, *i.e.* manifestation of anaemia in the late or early stage of the developing myeloid leukaemia in RFM/Un mice, using radio-iron incorporation in blood as an indicator.

### MATERIALS AND METHODS

The details of the mice and leukaemia line used have been given elsewhere (Tanaka and Lajtha, 1969; Tanaka and Craig, 1970). Briefly, the leukaemic cell line has been maintained at 7 to 10 day intervals by intravenous injections of $10^5$–$10^6$ leukaemic spleen cells in suspensions.

*Assay of $^{59}Fe$ incorporation in blood.*—Suspensions of leukaemic spleen cells from mice with advanced leukaemia were injected into groups of mice, $10^5$ cells intravenously. Groups of these mice were then injected daily with $^{59}$Fe (0·5 $\mu$Ci per mouse, intramuscularly) as ferric citrate (average specific activity 20 $\mu$Ci/$\mu$g.). Twenty-four and 48 hours after iron injections, the animals were killed and $^{59}$Fe concentrations in the blood were determined on 0·2 ml. of whole blood by a scintillation counter.

*Exorepopulation.*—This technique was described originally by Hodgson (1962a, 1962b) and estimates the size of haematopoietic stem-cells in normal mice. Its application to the leukaemia system was described elsewhere (Tanaka, Craig and Lajtha, 1970). Leukaemic spleen cell suspensions were injected into groups of mice, $10^5$ cells intravenously. These " donor " mice were killed daily from day 1 to

* Present address: Department of Experimental Pathology and Cancer Research, School of Medicine, University of Leeds, Leeds 2.

day 12, and appropriate dilutions of the bone marrow or spleen cell suspensions were injected into total body irradiated (900 rad) " recipient " mice. Seven days after irradiation and injection, $^{59}Fe$ (0·5 $\mu$Ci per mouse) was injected into a group of recipients intramuscularly. On day 9, *i.e.* 48 hours after iron injections, 0·2 ml. of blood was collected and $^{59}Fe$ uptake was determined.

*Autorepopulation.*—This technique has been applied to stem-cell recovery after irradiation (Porteous and Lajtha, 1966). Leukaemic spleen cell suspensions of appropriate dilutions were injected into groups of mice as before. Groups of mice were irradiated (700 rad) with both femora- or one femur-shielded with 7 mm. long and 4 mm. thick lead plate (Fig. 1). Seven days later $^{59}Fe$ was injected, and mice were killed for assessment of iron-uptake 48 hours later.

FIG. 1.—An experimental scheme of the autorepopulation technique applied to leukaemia.

### RESULTS

*Assay of $^{59}Fe$ incorporation in blood.*—The 48-hour iron-uptake remained at the normal level of 41% up to day 6 after inoculation of leukaemic cells and then gradually declined to a level of 26% by day 10 (Fig. 2). Similarly, the 24-hour iron-uptake started to decrease around day 6 from the normal level of 32% to 17% by day 10.

*Exorepopulation.*—Bone marrow cell grafting: In a group given $10^6$ cells from donors inoculated with leukaemic cells, iron-uptake started to decline from the normal level of 15·5% to 8% on day 7 of the leukaemia (Fig. 3a). However, from day 9 of the leukaemia onwards, none of the treated recipients survived the 9-days post-treatment required for assaying iron-uptake. In a group given $5 \times 10^5$ cells, iron-uptake decreased after day 8 although the majority of recipients were not able

FIG. 2.—$^{59}$Fe uptake (at 24 and 48 hours after iron injections) in peripheral blood of leukaemic cell-inoculated mice. (Shaded areas represent the normal ranges.)

to survive for 9 days required for assay. In groups given $10^4$ and $10^5$ cells, iron-uptake virtually remained at the same level throughout the leukaemia development, 0·45% and 1·3% respectively.

Spleen cell grafting: In a group given $6 \times 10^6$ cells from donors inoculated with leukaemic cells, iron-uptake decreased to 14% from the normal level of 21% on day 8 of the leukaemia (Fig. 3b). As seen in the bone marrow cell grafting, from day 9 of the leukaemia onwards treated recipients died. In groups given $6 \times 10^5$ and $3 \times 10^6$ cells, iron-uptake remained the same at the early leukaemic stage, 2·2% and 12·5% respectively. The majority of the recipients in a group given $3 \times 10^6$ cells were at too advance a stage by day 9.

*Autorepopulation.*—One femur-shielded: Four groups of animals were injected with leukaemic spleen cell suspensions consisting of $6 \times 10^4$, $6{\cdot}8 \times 10^4$, $2{\cdot}5 \times 10^5$ and $2{\cdot}8 \times 10^5$ cells. Iron-uptake started to decrease in all on day 7 of the leukaemia from 4·5% to 2·5% (Fig. 4).

Both femora-shielded: Three groups of animals were injected with leukaemic

FIG. 3a.—$^{59}$Fe uptake in peripheral blood of irradiated and *bone-marrow cell*-grafted mice (exorepopulation).

cells consisting of $6{\cdot}8 \times 10^4$, $8 \times 10^4$ and $1{\cdot}25 \times 10^5$ cells. Iron-uptake began to decline in all on day 7 from 10·5% to 6% by day 9 (Fig. 4).

### DISCUSSION

The methods described to assess haematopoiesis during the leukaemia development have admittedly several disadvantages, especially for studies involving malignant cell populations. Exorepopulation: The lowest cell inoculum size for " proper " iron-uptake, *i.e.* more than 5% of total radio-iron injected, is limited to about $5 \times 10^4$ cells (Hodgson, 1962a, 1962b). This is unquestionably a large inoculum, particularly in the late stage of any type of leukaemias. With such a large dose of malignant cells, the treated animals certainly cannot survive for a sufficient period required for iron-uptake as well as colony assay. In contrast, $10^2$ to $10^3$ cell inoculum sizes are very useful ranges for assessment of leukaemia colonies, as seen in lymphoma (Bruce and van der Gaag, 1963) or lymphocytic leukaemia (Wodinsky *et al.*, 1967) or myeloid leukaemia (Tanaka and Lajtha, 1969).

FIG. 3b.—$^{59}$Fe uptake in peripheral blood of irradiated and *spleen cell*-grafted mice (exorepopulation). (Shaded areas represent the normal ranges and figures cell inoculum sizes.)

Autorepopulation: In experiments with either one femur- or both femora-shielded, the treated mice were not able to survive beyond day 10 after injections of the leukaemic cells. It should be remembered that the mice in the day 9 group were kept for another 9 days for $^{59}$Fe assay, *i.e.* they actually survived a total of 18 days after leukaemic cell injections. This was the longest survival time after injections with the cell inoculum sizes used. Nevertheless, the autorepopulation technique, especially with both femora-shielded, was a more ideal measurement of iron-uptake than exorepopulation. This coincides with the view that " the autorepopulation test is a fair picture of the animal's capability for haemopoiesis " (Lajtha *et al.*, 1969).

Due to the several technical limitations, the erythropoietic response during the leukaemia development cannot be explored fully up to the very advanced stage.

FIG. 4.—$^{59}$Fe uptake in peripheral blood of leukaemic cell-injected and irradiated mice with one femur- or both femora-shielded (autorepopulation). (Shaded areas represent the normal ranges. The symbols indicate four and three different dilutions of leukaemic spleen cell suspensions used in one femur- and both femora-shielded mice respectively.)

However, these techniques as a whole do suggest that erythropoiesis decreases during the leukaemic process owing to a decline in numbers of the normal erythropoietic cell precursors. This is further elucidated by the response pattern in numbers of the normal haemopoietic colony formers from the bone marrow and spleen to an increasing proportion of the myeloid leukaemia colony-formers recoverable from these organs (Tanaka *et al.*, 1970). Yet, the progress of anaemia is rather slow, and apparently erythropoiesis is not interfered with until the late stage of the leukaemic process. This may be due to the compensatory erythropoiesis in the spleen (Handler and Handler, 1970) as well as in the liver. The cause for decreasing erythropoiesis is most likely due to a mechanical factor, *i.e.* replacement of erythropoietic tissue by the proliferating leukaemic mass; a similar mechanism suggested in rats (Handler *et al.*, 1968) and reported in man (Wassi and Block, 1961).

### REFERENCES

Brodsky, I., Dennis, L. H. and Kahn, S. B.—(1966) *Cancer Res.*, **26**, 1887.

Bruce, W. R. and van der Gaag, H.—(1963) *Nature, Lond.*, **199**, 79.

Handler, E. E. and Handler, E. S.—(1970) *J. Reticuloendothel. Soc.*, **7**, 328.

Handler, E. E., Handler, E. S. and Schaffer, A. E.—(1968) *J. Reticuloendothel. Soc.*, **5**, 445.

Helyer, B. J. and Howie, J. B.—(1958) *Rep. Br. Emp. Cancer Campn*, **36**, 458.

Hodgson, G. S.—(1962a) *Acta physiol. latinoam.*, **12**, 365.—(1962b) *Blood*, **19**, 460.

Lajtha, L. G., Pozzi, L. V., Schofield, R. and Fox, M.—(1969) *Cell Tissue Kinetics*, **2**, 39.

Ludwig, F. C., Bostick, W. L. and Epling, M. L.—(1964) *Cancer Res.*, **24**, 1308.

Mirand, E. A., Prentice, T. C., Hoffman, J. C. and Grace, J. T.—(1961) *Proc. Soc. exp. Biol. Med.*, **106**, 423.

Porteous, D. D. and Lajtha, L. G.—(1966) *Br. J. Haemat.*, **12**, 177.

Russell, E. S. and Bernstein, S. E.—(1966) 'Blood and Blood Formation'. In 'Biology of the Laboratory Mouse', 2nd edition. Edited by E. L. Green. New York (McGraw-Hill), Chapter 17.

Tanaka, T. and Craig, A. W.—(1970) *Eur. J. clin. biol. Res.*, **15**, 505.

Tanaka, T., Craig, A. W. and Lajtha, L. G.—(1970) *Br. J. Cancer*, **24**, 138.

Tanaka, T. and Lajtha, L. G.—(1969) *Br. J. Cancer*, **23**, 197.

Wassi, P. and Block, M.—(1961) *Blood*, **17**, 597.

Wodinsky, I., Swiniarski, J. and Kensler, C. J.—(1967) *Cancer Chemother. Rep.*, **51**, 415.